# Invasive Serotype 35B Pneumococci Including an Expanding Serotype Switch Lineage, United States, 2015–2016

**DOI:** 10.3201/eid2306.170071

**Published:** 2017-06

**Authors:** Sopio Chochua, Benjamin J. Metcalf, Zhongya Li, Hollis Walker, Theresa Tran, Lesley McGee, Bernard Beall

**Affiliations:** Centers for Disease Control and Prevention, Atlanta, Georgia, USA

**Keywords:** serotype switch, serotype expansion, switch lineage, invasive pneumococcal disease, pneumococcal conjugate, pneumococci, bacteria, vaccines, pneumococcal 7-valent conjugate vaccine, pneumococcal 13-valent conjugate vaccine, penicillin-binding protein types, serotype 35B, surveillance, recombination, respiratory infections, United States

## Abstract

We used whole-genome sequencing to characterize 199 nonvaccine serotype 35B pneumococcal strains that caused invasive pneumococcal disease (IPD) in the United States during 2015–2016 and related these findings to previous serotype 35B IPD data obtained by Active Bacterial Core surveillance. Penicillin-nonsusceptible 35B IPD increased during post–pneumococcal 7-valent conjugate vaccine years (2001–2009) and increased further after implementation of pneumococcal 13-valent conjugate vaccine in 2010. This increase was caused primarily by the 35B/sequence type (ST) 558 lineage. 35B/ST558 and vaccine serotype 9V/ST156 lineages were implicated as *cps35B* donor and recipient, respectively, for a single capsular switch event that generated emergent 35B/ST156 progeny in 6 states during 2015–2016. Three additional capsular switch 35B variants were identified, 2 of which also involved 35B/ST558 as *cps35B* donor. Spread of 35B/ST156 is of concern in view of past global predominance of pathogenic ST156 vaccine serotype strains. Protection against serotype 35B should be considered in next-generation pneumococcal vaccines.

Although the dramatic protective effect of the pneumococcal 7-valent conjugate vaccine (PCV7) against invasive pneumococcal disease (IPD) persisted a full decade after its introduction in the United States in 2000, the emergence of 19A and other non-PCV7 serotypes reduced the overall benefit (*1**,**2*). Before PCV7 implementation, we observed only 2 different 35B lineages within Active Bacterial Core surveillance (ABCs) (*3*), a population-based, multistate program that assesses the effect of invasive bacterial infections and is part of the Emerging Infections Program network of the Centers for Disease Control and Prevention (CDC; Atlanta, GA, USA) (http://www.cdc.gov/abcs/index.html). Both lineages were relatively rare causes of IPD but were geographically widespread in the United States before and after PCV7 introduction (*3*). One 35B lineage was antimicrobial-susceptible and multilocus sequence type (MLST) 452 (35B/ST452), and the second strain was penicillin-nonsusceptible 35B/ST558. During 1995–2001, the penicillin-nonsusceptible 35B/ST558 lineage, which had resistant MICs of 0.25–2.0 μg/mL, accounted for 69% of serotype 35B ABCs isolates (*3*). During 1999–2007 in the United States, the proportion of penicillin-nonsusceptible IPD isolates within serotype 35B increased to 84%; 35B/ST558 accounted for this increase (*4*). Consistent with this observation was a 9-fold increase in carriage of 35B/ST558 in young children in the Atlanta, Georgia, area (*5*).

After introduction of the 13-valent conjugate vaccine (PCV13) in 2010, 35B became the most common serotype in ABCs, associated with MICs >2 μg/mL for penicillin and amoxicillin in pediatric isolates (*6*) and in the adult population (B. Beall, unpub. data). Consistent with its status as a major cause of IPD in the post-PCV13 era, the 35B/ST558 lineage is currently commonly found in disease and asymptomatic pneumococcal carriage in many countries (*7**–**11*).

We provide a whole-genome sequence (WGS) pipeline−based resolution and description of current 35B lineages within current ABCs surveillance (*6**,**12*), including an invasive 35B variant of the historically successful ST156 lineage. Recently, we identified 2 different 35B isolates recovered during 2009 and 2012 that each appeared to have arisen through a unique capsular switch event involving the same 2 parental strains. This observation was made on the basis of the penicillin-binding protein (PBP) gene types flanking the 35B biosynthetic locus (*cps35B*) in each of the variants (*6*).

Only the 35B/ST156 variant detected during 2012 has emerged and has been detected within 6 states. ST156 has a remarkable history of conjugate vaccine evasion. Formerly the primary genotype of PCV7 serotype 9V in the United States during the preconjugate vaccine era (*13*), 9V greatly decreased after PCV7 implementation (*1*) and was partially replaced by resistant 19A/ST156 (*14*). To verify that these isolates originated from a single recombinational serotype switch event involving 35B/ST558 and 9V/ST156 parental strains, we analyzed regions flanking the *cps35B* locus during 2015–2016, 35B/ST156 progeny and the original strain detected during 2012.

## Methods

### Isolates

The surveillance population of ABCs is ≈32 million persons in 10 states (http://www.cdc.gov/pneumococcal/surveillance.html). Serotype 35B IPD isolates described include 132 recovered during 2015 and 67 recovered during ABCs in 2016 (Table 1, https://wwwnc.cdc.gov/EID/article/23/6/17-0071-T1.htm). The listing of 35B isolates from 2016 is incomplete because we typically receive all ABCs isolates recovered during a given year by the following summer. Relevant ST156 lineage isolates of other serotypes recovered during this and previous periods are shown in Table 2. Total numbers of ABCs 35B isolates recovered during 1999–2015 and categorized by patient age, penicillin MIC, and IPD incidence are shown in Table 3.

**Table 1:** WGS pipeline features of 199 invasive serotype 35B pneumococcal isolates, United States, 2015–2016 and 2 35B ST156 lineage isolates obtained during 2009 and 2012*

CC (no.)†	ST	No.	PBP type‡	Non- PBP resistance determinants§	Antimicrobial resistance phenotype, μg/mL¶										States
					Pen	Amo	Tax	Cft	Cfx	Mer	Ery	Cli + Tet	Cot	Fq#	
**156 (21)**	156	15**	4:**12**:7	*mef*, *folA*I100L, *folPins178*	2	4	1	1	>2	**1**	R	S	R	S	CT, GA, MN, NY, TN, MD
	10174	1††	4:7:**18**	*mef*, *folA*I100L, *folPins178*	2	1	1	1	>2	0.5	R	S	R	S	NM
	11584	2	4:**12**:7	*mef*, *folA*I100L, *folPins178*	2	4	1	1	>2	**1**	R	S	R	S	TN
	9910	1	4:**12**:7	*mef*, *folA*I100L, *folPins178*	2	4	1	1	>2	**1**	R	S	R	S	TN
	162	1	0:0:0	None	<0.03	<0.03	<0.06	<0.03	<0.5	<0.06	S	S	S	S	NM
	12921	1	4:**11**:7	*mef, folA*I100L, *folPins178*	2	**8**	1	1	>2	**1**	R	S	R	S	TN
**63 (1)**	11818	1	4:31:114	*ermB, tetM, folPins195*	1	4	1	0.5	>2	0.5	R	R	I	S	MD
**1092 (1)**	1092	1	6:7:36	*folA*I100L, *folPins195*	1	1	0.5	1	>2	0.25	S	S	R	S	NM
452 (10)	452	8	0:0:0	None	<0.03	<0.03	<0.06	<0.03	<0.5	<0.06	S	S	S	S	CA, CT, NM, OR
	452	1	0:0:0	*mef*	<0.03	<0.03	<0.06	<0.03	<0.5	<0.06	R	S	S	S	MD
	452	1	0:0:**148**	None	**0.06**	≤0.03	**0.12**	**0.12**	<0.5	<0.06	S	S	S	S	CO
558 (168)	558	104	4:7:7	*mef*	2	4	1	1	>2	0.5	R	S	S	S	CA, CO, CT, GA,MD,MN, NM, NY, TN
	558	1	4:7:7	*mef,* ParC-D83Y, GyrA-S81Y	2	4	1	1	>2	0.5	R	S	S	R	TN
	558	1	4:7:7	*mef,* ParC-D83Y	2	4	1	1	>2	0.5	R	S	S	I	MD
	558	1	4:7:7	*mef,* ParC-S79F	2	4	1	1	>2	0.5	R	S	S	I	CT
	558	1	4:7:7	*mef, folPins186*	2	4	1	1	>2	0.5	R	S	I	S	CA
	558	22	4:7:7	None	2	4	1	1	>2	0.5	S	S	S	S	CA, CO, CT, GA, MD, MN, NM,TN
	558	2	4:7:7	*folA*I100L	2	4	1	1	>2	0.5	S	S	I	S	GA, MD
	558	1	4:7:7	*folPins173*	2	4	1	1	>2	0.5	S	S	I	S	TN
	558	1	4:7:7	*folPins189*	2	4	1	1	>2	0.5	S	S	I	S	MD
	558	1	4:7:7	*folPins195*	2	4	1	1	>2	0.5	S	S	I	S	NM
	558	1	4:7:7	*folAI100L, folPins180*	2	4	1	1	>2	0.5	S	S	R	S	CO
	558	1	4:7:7	*folAI100L, folPins169*	2	4	1	4	>2	0.5	S	S	R	S	CO
	558	1	4:7:28	*mef*	0.12	<0.03	<0.06	0.06	<0.5	<0.06	R	S	S	S	CO
	558	1	4:7:112	*mef*	2	2	1	1	>2	0.5	R	S	S	S	TN
	558	1	4:7:133	*mef*	2	2	1	1	>2	0.5	R	S	S	S	GA
	558	2	4:1**20**:7	*mef*	2	4	1	1	>2	1	R	S	S	S	CT
	558	1	4:123:7	*mef*	2	4	1	1	>2	0.5	R	S	S	S	NY
	558	1	102:7:7	*mef*	2	2	1	1	>2	0.5	R	S	S	S	CT
	558	1	106:7:7	None	1	1	0.5	0.5	>2	0.5	S	S	S	S	MD
	558	1	4:135:7	*folAI100L, folPins169*	2	4	1	4	>2	0.5	S	S	R	S	CO
	558	1	4:139:7	*mef*	2	4	1	4	>2	0.5	R	S	S	S	MN
	6961	1	4:7:7	None	2	4	1	1	>2	0.5	S	S	S	S	MD
	7486	1	4:7:7	*mef*	2	4	1	1	>2	0.5	R	S	S	S	TN
	7487	1	4:**49**:7	None	2	**8**	1	1	>2	**1**	S	S	S	S	CT
	10493	6	4:7:7	*mef*	2	4	1	1	>2	0.5	R	S	S	S	CT, GA, MD, NM
	10493	1	4:7:7	*mef*, *folA*I100L	2	4	1	1	>2	0.5	R	S	I	S	NM
	11250	1	4:**14**:7	*folPins169*	2	**8**	1	1	>2	**1**	S	S	I	S	GA
	11254	2	4:7:7	*folA*I100L, *folPins169*	2	4	1	1	>2	0.5	R	S	R	S	CO, MD
	11603	1	4:7:7	*mef*	2	4	1	1	>2	0.5	R	S	S	S	CT
	11604	1	4:7:7	*mef*	2	4	1	1	>2	0.5	R	S	S	S	MD
	11606	1	4:7:7	None	2	4	1	1	>2	0.5	S	S	S	S	CO
	11866	1	4:7:7	*mef*	2	4	1	1	>2	0.5	R	S	S	S	GA
	12854	1	4:7:7	*mef*	2	4	1	1	>2	0.5	R	S	S	S	GA
	12883	1	4:7:7	*20L*	2	4	1	1	>2	0.5	S	S	I	S	MN
	12885	1	4:7:7	*mef*	2	4	1	1	>2	0.5	R	S	S	S	CT
	12922	1	4:142:7	None	4	8	1	2	>2	>1	S	S	S	S	MD

*All isolates were positive for pilus PI-type 1 and negative for pilus PI-type 2. CC, clonal complex; I, intermediate resistance; MLST, multilocus sequence type; PBP, penicillin-binding protein; R, resistant; S, susceptible; ST, sequence type; WGS, whole-genome sequencing. †CCs that probably arose through serotype switching are indicated in bold. ‡MIC correlates for PBP types (http://www.cdc.gov/streptlab/mic-tables.html (*15*). Variant PBPs potentially contributing to increased MICs relative to 4:7:7 or 0:0:0 are indicated in bold. Three isolates yielded only partial PBP types because of genomic assembly error, but were assigned PBP type 4:7:7 because of partial identity and consistent phenotypic β-lactam MIC profile. §For a description of WGS-based bioinformatic pipeline for deduction of all features shown, see Gertz et al (*4*) and Desai et al. (*7*). For a description of *folP* insertions (*folPins169*), see Figure 1 in Gertz et al. (*4*). ¶Predicted MICs for β-lactam antimicrobial drugs were based on transpeptidase domain sequences of PBPs 1a, 2b, and 2x (http://www.cdc.gov/streplab/mic-tables.html). For penicillin (meningitis only), nonsusceptible is considered >0.12 μg/mL (*16*). Currently applied clinical cutoffs are also provided for the other 5 β-lactams shown (*16*). Where shown, I, R, and S correspond to breakpoint MIC values (*16*). Increased MICs potentially attributable to PBP type alterations are indicated in bold. Amo, amoxicillin; Cft, ceftriaxone; Cfx, cefuroxime; Cli, clindamycin; Cot, cotrimoxazole; Ery, erythromycin; Fq, fluoroquinolones levofloxacin and ciprofloxacin; Mer, meropenem; Pen, penicillin; Tax, cefotaxime. #MIC of 4 μg/mL for ciprofloxacin is considered intermediate resistance. **Includes single putative progenitor strain (2012221165) isolated from a 4-y-old child during 2012 (Figure 2). ††2009219987 was isolated from an infant in New Mexico during 2009 (Figure 2).

**Table 2:** Nonserotype 35B isolates of ST156 lineage included in study of penicillin-nonsusceptible 35B pneumococcal isolates causing IPD, United States, 1998–2015*

Serotype/ MLST type (no.)†	No.	PBP type‡	Non-PBP resistance determinants§	Antimicrobial resistance phenotype, MIC, μg/mL¶										State (year isolated)
				Pen	Amo	Tax	Cft	Cfx	Mer	Ery	Cli + Tet	Cot	Fq	
9V/156 (25)	12	15:12:18	*folA*I100L, *folPins178*	4	2	1	2	>2	0.5	S	S	R	S	CA, GA, MD, MN, NY, TN (1998–1999)
	9	15:12:18	*mef, folA*I100, *folPins178*	4	2	1	2	>2	0.5	R	S	R	S	CA, CT, MD, MN, OR, TN (1998,1999, 2015)
	2	15:12:18	*ermB, tetM*, *folA*I100L, *folAins178*	4	2	1	2	>2	0.5	R	R	R	S	CA (2015)
	1	15:12:18	*mef, tetM, folA*I100L, *folPins178*	4	2	1	2	>2	0.5	R	S	R	S	CT (2015)
	1	15:12:228	*Mef, folA*I100L, *folPins178*	4	2	1	2	>2	0.5	R	S	R	S	MD (2016)
**19A/156 (4)**	3	29:12:26	*mef, folA*I100L, *folAins189*	4	2	8	4	>2	0.5	R	S	R	S	CA,GA (2009)
	1	8:12:36	*mef, folA*I100L, *folAins189*	1	2	0.5	0.5	2	0.25	R	S	R	S	GA (2015)
**13/156 (1)**	1	15:12:173	*folA*I100L, *folPins178*	1	1	0.25	0.25	1	0.5	S	S	R	S	TN (2015)
**31/156 (1)**	1	15:12:18	*mef*, *folA*I100L, *folPins178*	4	2	1	2	>2	0.5	R	S	R	S	MN (2015)

*All isolates were positive for pilus PI-type 1 and negative for pilus PI-type 2. IPD, invasive pneumococcal disease; MLST, multilocus sequence type; PBP, penicillin-binding protein; R, resistant; S, susceptible; ST, sequence type. †Types that probably arose through serotype switching are indicated in bold. ‡See Li et al. (*15*) and MIC correlates for PBP types (http://www.cdc.gov/streplab/mic-tables.html). §For a description of WGS-based bioinformatic pipeline for deduction of all features shown, see Metcalf et al. (*6,12)*. For a description of *folP* insertions (*folPins178*, *folP189*), see Figure 1 in Metcalf et al. (*12*). ¶Predicted MICs for β-lactam antimicrobial drugs were based on transpeptidase domain sequences of PBPs 1a, 2b, and 2x (http://www.cdc.gov/streplab/mic-tables.html). For penicillin (meningitis only), nonsusceptible is considered >0.12 μg/mL (*16*). Currently applied clinical cutoffs are also provided for the other 5 β-lactams shown (*16*). Where shown, R and S correspond to breakpoint MIC values (*16*). Amo, amoxicillin; Cft, ceftriaxone; Cfx, cefuroxime; Cli, clindamycin; Cot, cotrimoxazole; Ery, erythromycin; Fq, fluoroquinolones levofloxacin and ciprofloxacin; Mer, meropenem; Pen, penicillin; Tax, cefotaxime.

**Table 3:** Annual incidence and proportions of penicillin-nonsusceptible 35B pneumococcal isolates causing IPD, United States, 1999–2015*

Year	Surveillance population	% CIS	No. 35B isolates from patients by age, y		Relative incidence of 35B IPD†	Pen MIC, μg/mL			*penNS *35B isolates/*penS* 35B isolates
			<5	≥5		>2	0.12−1	<0.06	
1998	17,383,935	86.1	3	18	1.40	9	5	7	2.0
1999	18,550,681	87.0	2	18	1.24	8	3	9	1.2
2000	19,821,607	86.3	4	21	1.46	7	9	9	1.8
2001	22,479,308	88.1	2	40	2.12	14	18	10	3.2
2002	25,051,246	87.6	2	34	1.64	20	8	8	3.5
2003	25,264,246	91.4	11	49	2.56	22	26	11	4.4
2004	27,419,898	87.9	15	69	3.49	44	25	15	4.6
2005	27,816,784	89.5	11	57	2.73	35	18	15	3.5
2006	28,204,455	86.7	1	65	2.70	37	15	14	3.7
2007	28,579,312	87.5	5	83	3.52	51	23	14	5.3
2008	28,856,774	86.7	12	80	3.68	62	16	14	5.6
2009	29,206,528	89.8	8	70	2.97	45	16	17	3.6
2010	29,757,552	90.2	4	67	2.65	52	14	5	13.2
2011	30,075,050	90.3	11	77	3.28	71	7	10	7.8
2012	30,356,544	90.6	13	101	4.14	94	14	6	18.0
2013	30,604,240	88.7	15	114	4.75	94	25	10	11.9
2014	31,328,211	88.2	16	116	4.78	104	22	6	21.0
2015	31,977,800	92.0	10	121	4.45	101	24	6	20.8

*CIS, case isolates serotyped; IPD, invasive pneumococcal disease. †Estimated cases/million = total 35Bs x 100/% CIS surveillance population/1,000,000.

### WGS and WGS-Based Predictions

Library construction and sequencing was performed as described (*12*). WGS accessions for all 199 serotype 35B isolates from 2015–2016, two previous 35B switch strains from previous years, and relevant strains of other serotypes of ST156 from previous years are provided (Technical Appendix Table). WGS pipeline data and quality metrics for all isolates are also provided (Technical Appendix Table). Capsular serotypes, antimicrobial genotypes/phenotypes, MLST, sequence type (ST), and pili (presence or absence) for year 2015–2016 isolates were deduced through our bioinformatics pipeline (*6**,**12**,**15*).

### Phylogeny

Paired-end fastq files were trimmed with Cutadapt version 1.8.1 (*17*), and draft genome assemblies were constructed by using VelvetOptimiser version 2.2.5 with an optimal kmer value calculated by using VelvetK (*18*). Core genome single-nucleotide polymorphism (SNP) identification and alignment were performed by using kSNP3.0 (*19*). A maximum-likelihood phylogenetic tree was generated from the core SNP alignment by using RaxML version 7.3.0 (*20*). RaxML was run with an ASC_GTRGAMMA DNA substitution model and used the Lewis method for ascertainment bias correction. Node support was assessed by using 500 bootstrap replicates.

### Conventional MIC Testing and Serotyping

Serotype 35B isolates recovered during 2015 were subjected to conventional broth dilution testing for determination of antimicrobial MICs. A selection of these isolates were also subjected to conventional serotyping by using CDC typing antisera as described (*6*).

### Statistical Analyses

A χ^2^ test was performed to evaluate differences among groups. This test was performed by using OpenEpi Version 3.01 (http://www.openepi.com/Menu/OE_Menu.htm).

## Results

### Increase in Penicillin-Nonsusceptible 35B during the Conjugate Vaccine Era

During 1998–2001, penicillin-nonsusceptible 35B accounted for 67.6% (108) of serotype 35B ABCs isolates (Table 3). During 2002–2015, the proportion of penicillin-nonsusceptible IPD isolates with serotype 35B increased to 87.7% (1,237; p<0.001).

### Population Snapshot of Ongoing ABCs for 35B IPD, 2015–2016

**Figure 1:**
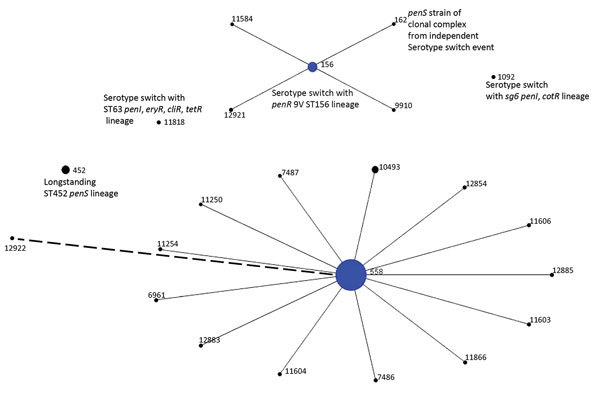
Population snapshot of 199 serotype 35B pneumococcal isolates obtained by ongoing Active Bacterial Core surveillance, United States, 2015–2016, configured by using eBURST (*21*). Diameters are proportional to number of isolates. Solid lines indicate single-locus variants, and the single dashed line indicates a double-locus variant of ST558. ST, sequence type.

Among 2,710 IPD isolates obtained during 2015 and subjected to WGS, 132 (4.9%) were serotype 35B. Of 1,528 IPD isolates recovered from partial year 2016 IPD surveillance, 67 (4.4%) were serotype 35B. Most (168/199) of these isolates belonged to penicillin-nonsusceptible clonal complex (CC) 558 (168 isolates) and CC156 (21 isolates) (Figure 1; Table 1). Serotype 35B CC558 and CC156 isolates of all serotypes discussed were uniformly positive for the *rrgA* gene (Tables 1, 2), which encodes a pilus subunit that functions in epithelial adhesion (*22*). Ten isolates of long-standing penicillin-susceptible 35B/ST452 (*3*) were also recovered. Single 35B isolates were identified of ST1092, a lineage of conjugate vaccine serotypes 6A and 6B (see http://pubmlst.org/spneumoniae/), and of ST11818 (highly related to 15A/ST63), an antimicrobial-resistant nonvaccine serotype lineage that has increased in the post–conjugate vaccine era (*4*).

### CC558 (35B/CC558)

Of 168 35B/CC558 isolates obtained, 147 were ST558, 20 were single-locus variants (SLVs) of ST558 corresponding to 13 STs, and 1 was a double-locus variant (Figure 1; Table 1). Within CC558, only ST558 had SLVs, which is consistent with initial successful establishment of 35B/ST558 in its ecologic niche and subsequent rare shedding of closely related SLVs (*21*).

The increased incidence of 35B IPD during the post-PCV7 period (2.1–3.7 cases/million population during 2001–2009 vs. 1.2–1.3 cases/million population during 1998–1999) and the post-PCV13 period (3.3–4.8 cases/million population during 2011–2015), combined with the consistent trend of markedly increased proportions of penicillin-nonsusceptible 35B IPD isolates throughout the conjugate vaccine era (Table 2), is consistent with reported increased 35B/ST558 in IPD and carriage (*4**–**11*). ABCs surveillance sites increased after 2000, but 35B IPD incidence calculations did not vary whether including the expanded surveillance sites or by using only continuously participating ABCs sites during 1998–2015.

### CC156 (35B/CC156)

We analyzed PBP types (*6**,**12**,**15*) of 35B/ST558 (4:7:7) and 35B/ST156 (4:12:7) isolates. These PBP amino acid sequence types are used for predicting β-lactam MICs and correspond to PBP transpeptidase domains from PBP1a, PBP2b and PBP2x, respectively. PBP genes *pbp1a* and *pbp2x* flank opposite ends of the capsular biosynthetic locus and are sometimes cotransferred during serotype switching events (*6**,**23**–**25*). The 35B/ST558 lineage has been nearly exclusively associated with PBP type 4:7:7 among isolates obtained since 1998, and the serotype 9V/ST156 lineage is similarly highly associated with PBP type 15:12:18 (*6**,**15*). However, 9V/ST156 is rare among IPD isolates in the post-PCV7 period.

**Figure 2:**
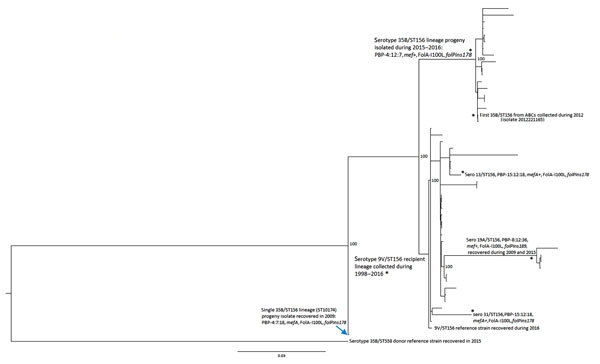
Phylogenetic analysis of potential recipient and serotype switch pneumococcal progeny strains within the ST56 lineage based upon a total alignment of 10,409 core single-nucleotide polymorphisms, United States, 2015–2016. All 20 serotype 35B progeny shown were recovered through Active Bacterial Core surveillance, and all but 2 indicated strains were obtained during 2015–2016. Isolate features are depicted for the 2 major nodes, with exceptions indicated by asterisks within the tree. Bootstrap values are indicated at key nodes. The serotype 9V isolate that was used for the reference recipient sequences described in Figure 3 is indicated as the third isolate from bottom. All isolates above the 9V recipient reference within this major cluster, except where indicated, are also 9V/ST156. Three additional serotype switch ST156 strain types detected by Active Bacterial Core surveillance during 2015–2016 are indicated by asterisks (single isolates of serotypes 31 and 13 and 4 serotype 19A isolates). Scale bar indicates nucleotide substitutions per site.

**Figure 3:**
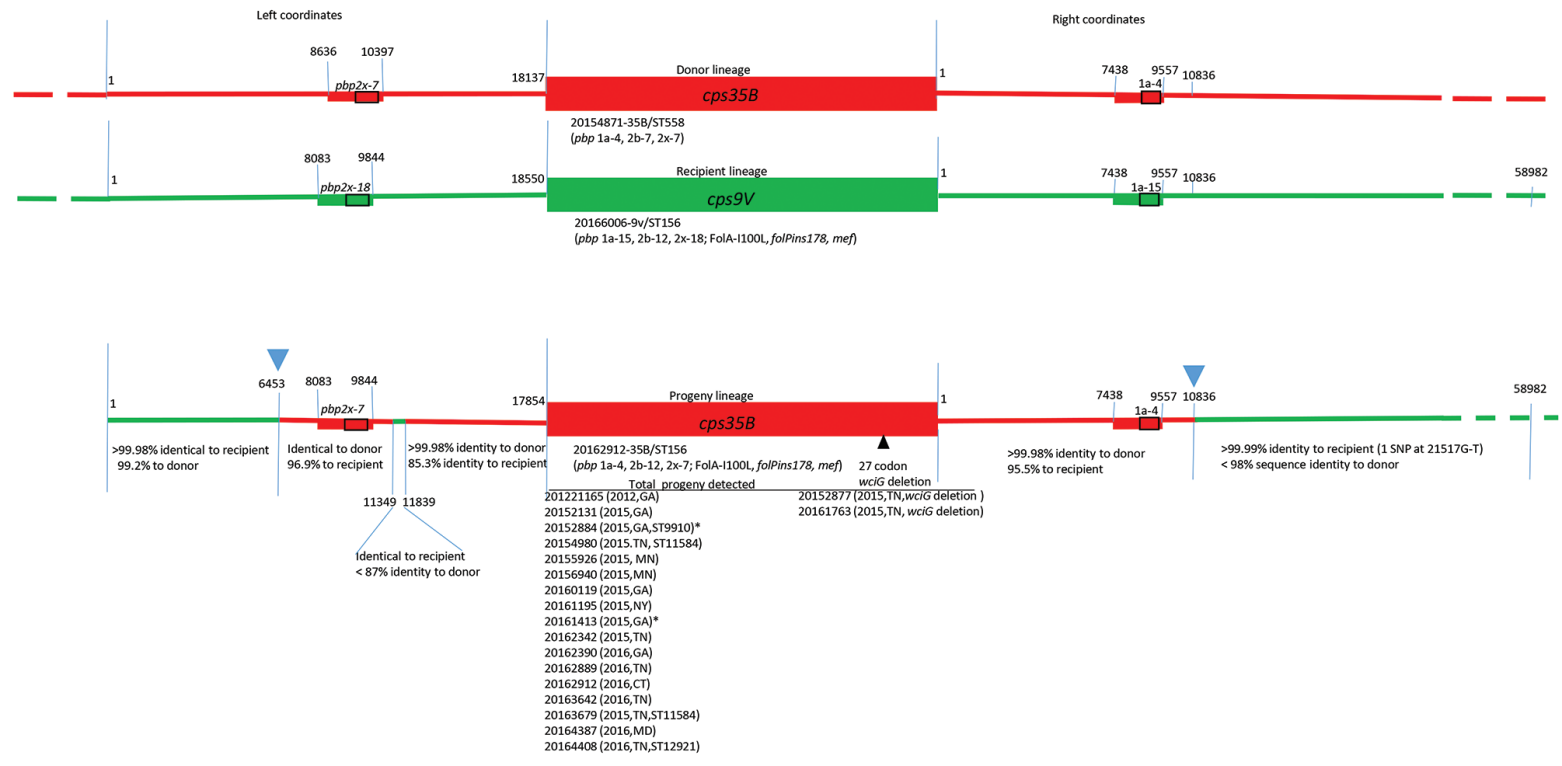
Diagrammatic representation of *cps* loci and adjacent regions from donor, recipient and progeny strains depicting serotype switch event for pneumococcal isolates, United States, 2015–2016. Red and green lines in progeny indicate regions of sequence identity or near identity (<2 single-nucleotide polymorphisms/10,000 bp) to the above corresponding donor and recipient sequences, respectively. Rectangles indicate relative locations of PBP gene types for *pbp2x* and *pbp1a.* Below each *cps* locus, a representative reference strain is indicated along with relevant features determined through a bioinformatics pipeline (MLST, PBP type, resistance markers). Junctions between donor and recipient sequences involved in the 2 single recombinational crossovers in the gene replacement event are indicated with blue arrowheads above the progeny diagram, although a single short internal region with sequence identity to the recipient nested within the donor fragment (left coordinates 11349–11839) is also present. Below each green or red segment of the progeny, the level of sequence identity to donor and recipient is provided. The list of each progeny strain, date of isolation, and state is provided. Where MLST is not ST156, its single locus variants (ST9910, ST11584, and ST12921) are included. Two exceptions indicating flanking post-switch recombination within left coordinates 1–6453 are indicated in isolates 20152884 and 20161413 (asterisks): isolate 20152884 had only 99.3%−99.5% identity to recipient and donor over bases 1–3715, and isolate 20161413 had only 99.5%–99.7% identity to recipient and donor over bases 1–2143. Two strains on the right indicate a post-switch deletion event within the *wciG* putative acetyltransferase gene, which putatively contributes to the acetylation pattern of the serotype 35B polysaccharide (*26*). MLST, multilocus sequence type; PBP, penicillin-binding protein; ST, sequence type.

In addition to the PBP2b-12 marker, *mef* gene, and FolA-I100L substitution, candidate *cps35B* recipient 9V/ST156 strains contain the 2-codon insertion designated *folPins178* (Table 2; Figures 2, 3). Such 1–2 codon *folP* insertions, which together with FolA-I100L confer cotrimoxazole resistance, are categorized by specific location of the insertion and specific sequence flanking the insertions (*12*). These genomic features are also found within the 35B/ST156 lineage isolates described (Table 1; Figures 2, 3), which are consistent with a 9V/ST156 (*mef*, FolA-I100L, *folPins178*) strain serving as the recipient strain for a 35B/ST558 *cps35B* donor strain (Figure 3). Another potential recipient strain present before and after PCV13 introduction was 19A/ST156 (*6**,**14*). However, this lineage is associated with the *folPins189* insertion (Table 2; Figure 2).

Both flanking *pbp* loci from 35B/ST558 were cotransferred with the *cps35B* locus to replace the *cps9V*, *pbp1a*-15 and *pbp2x*-18 determinants in the putative 9V/ST156 recipient, which resulted in PBP type 4:12:7 (Figure 3). This serotype switch progeny strain was obtained from a 4-year-old child during 2012 and is a potential progenitor of the current invasive 35B/CC156 lineage (isolate 2012221165) (Figures 2, 3). Antimicrobial resistance markers PBP2b-12, *mef*, FolA-I100L, and *folPins178*, combined with the close phylogenetic relatedness of the 9V/ST156 isolates (Figure 2), suggest that a member of this lineage served as the recipient parental strain for the 35B/ST156 clade isolated in ongoing ABCs during 2015–2016 (Figure 3).

Analysis of the regions flanking the *cps35B* locus for all 35B/ST156 lineage isolates obtained during 2015–2016 showed identical recombinational sites at bases 6,453 (left coordinate of progeny reference) (Figure 3) and 10,836 (right coordinate), which is clearly indicative of a single event within a 35B/ST156 ancestral strain of the 19 progeny shown (Figure 3). Thus, a double-crossover event replaced the recipient strain *cps9V* locus and flanking PBP markers (2x-18 and 1a-15) with the *cps35B* locus and its flanking PBP markers (2x-7 and 1a-4). On the basis of available strain data, the original progeny strain is predicted to have been an ST156 strain with the PBP type 4:12:7; a total of 14 of the 19 35B/ST156 lineage strains still shared these characteristics (Table 1). Five isolates are SLVs or differ in PBP2b type.

We detected the small (491 bp) segment (bases 11349–11839 of progeny) (Figure 3) of clearly recipient lineage origin within the left side of the major recombinational fragment. During a single–double crossover event that facilitates a serotype switch, additional independent double-crossover events appear to occur concurrently (*27*). However, these events probably do not occur simultaneously. It appears that the actual serotype switch event involved a shorter donor fragment bordering upon the right side of this small recipient lineage segment (base 11839), followed by a second double-crossover event that bordered upon the left side of the recipient lineage fragment (base 11349).

A single penicillin-susceptible 35B/ST162 (SLV of ST156) has the completely sensitive PBP type 0:0:0 (*6**,**12*) (Table 1). This strain arose through an independent serotype switch event that involved a penicillin-susceptible recipient strain.

### Postserotype Switch Event Diversification of 35B/ST156 Progeny

Five of the 19 35B/ST156 lineage progeny showed indications of genetic diversification that occurred after the capsular switch event. Four of these isolates have 1 of 3 SLV MLSTs of ST156 (ST9910, ST11584, and ST12921) (Table 1). Although 18 of the 19 strains were PBP type 4:12:7, the SLV ST12921 variant had PBP type 4:11:7. For this particular strain, it is probable that recombination with a highly penicillin-resistant ST320 strain, prevalent during the post-PCV7 era and having PBP type 13:11:16 (*6*), simultaneously replaced the *pbp2b* locus and flanking *ddl* sequence to change the PBP type to 4:11:7 and the MLST type to ST12921 through transfer of *ddl* with the resistance-conferring selectable *pbp2b* allele (*28*). We also observed an increased MIC for amoxicillin for this PBP type 4:11:7 strain compared with MICs for PBP type 4:12:7 strains (Table 1).

Two isolates (20152877 and 20161763) underwent a postswitch intra–*cps35B* gene deletion event within the *wciG* gene (Figure 3), which is predicted to encode an acetyltransferase (*26*). Although these 2 isolates were serotyped as 35B by using CDC typing antisera, they differed in reactivity with serologic factor 35a compared with the other 17 isolates of this lineage (Table 4). Typing antisera factors 29b and 35c are the CDC Quellung reagents definitive for serotype 35B. The original protocol (*29**,**30*) that CDC first followed for serogroup 35 resolution also used factor 35a along with factors 29b and 35c for identification of serotype 35B. We found that the 2 *wciG* deletion strains did not react with factor 35a, but the other 17 serotype 35B strains reacted strongly with factor 35a. These preliminary data is suggestive of a new serotype within serogroup 35 because this specific factor reactivity pattern has not been observed for serogroup 35 (*31*).

**Table 4:** Serologic comparison of 35B/ST156 lineage strains with CDC Quellung reagents for resolution of serogroup 35B invasive serotype pneumococci including an expanding serotype switch lineage, United States, 2015–2016*

Strain	Quellung factor				
	35b	29b	35c	42a	35a
*cps35B*†	−	+	+	−	+
*cps35B* (*wciG* deletion)‡	−	+	+	−	−

*CDC, Centers for Disease Control and Prevention; ST, sequence type; −, negative; +, positive. †Refers to 17 progeny resulting from recombination indicated in left column under progeny lineage diagram in Figure 3. ‡Refers to 2 indicated *wciG* deletion isolates in right column under progeny lineage diagram in Figure 3.

Further indication of chromosome-wide postswitch diversification of this single clade was shown in the left-flanking region of the *cps35B* locus. Two progeny strains showed diversification within the first 2–3.7 kb when compared with the other 17 progeny. The 6-kb region immediately to the left of base 1 in all of the progeny strains had <99.2% sequence identity with the most similar potential ST156 parental recipient strains that we analyzed. However, beyond this segment, progeny had sequence identity with the parental strain for >8 kb.

### 35B/ST156 Variant Lineages Arising through Separate Serotype Switch Events

The 9V/ST156 clade also appears likely to have served as the recipient for an independent serotype switch from the same 2 parental strains (Figure 3) which resulted in 35B/ST10174, an SLV of ST156 (Figure 2) obtained from an infant during 2009 (isolate 2009219987). This isolate differs in the flanking *pbp2x* marker and distal *pbp2b* marker (PBP type 4:7:18). Features of this variant have been described (*6*), and we have not obtained additional 35B isolates with these distinguishing features. A third serotype switch event involving a CC156 recipient strain is intuitive from the pipeline data, which indicate that the 35B SLV of ST162 is featured by the β-lactam–susceptible PBP type 0:0:0 (Table 1). ST162 has long been associated with penicillin-susceptible 9V strains (*13*) and more recently with PI-1 positive and penicillin-susceptible 23B, 15B, and 15C strains (*6*).

### Nonserotype 35B Variants of ST156

Single ST156 isolates of serotypes 31 and 13 were obtained during 2015 (Figure 2) and showed high relatedness to different 9V subdivisions. Again, the likely recipient background for the presumed capsular switch does not appear likely to involve the 19A/ST156 lineage (Figure 2), which was well-represented during the 2000s after PCV7 implementation (*6**,**14**,**32*).

### CCs of Remaining 35B Isolates Obtained during 2015–2016

Ten of the 12 isolates other than penicillin-nonsusceptible CC558 and CC156 (together composed of 187 isolates) were of the long-established penicillin-susceptible 35B/ST452 lineage (*3*), which decreased in proportion during the 2000s (*4*) (Figure 1; Table 3). The 2 remaining 35B isolates obtained during 2015–2016 also appear to have originated through serotype switching events involving a 35B/ST558 *cps35B* donor, as implicated by the presence of the PBP1a-4 or PBP2x-7 determinants flanking the *cps* loci of these 2 progeny strains (35B/ST11818 and 35B/ST1092) (Table 1). The 35B/ST11818 variant is an SLV of ST63 and has the same resistance features and accessory resistance genes (*ermB* and *tetM*) as the currently common 15A/ST63 clone (*4**,**6**,**32*). The 35B/ST1092 isolate is likely to have originated through serotype switching with a serogroup 6 recipient strain (*6*). The 14 remaining ST1092 isolates obtained during surveillance in 2015–2016 were serotype 6C. The 6 previously collected ST1092 IPD isolates (1999–2013) represented in our WGS collection are from serotype 6A, 6B, and 6C strains (*6*; B. Beall, unpub. data).

## Discussion

An increase of penicillin-nonsusceptible serotype 35B IPD and carriage caused by 35B/ST558 has been apparent in the United States since the introduction of PCV7 in 2000, and it has shown a major increase after PCV13 implementation (*4**–**8*). This finding is of concern because even strains that are rarely detected in IPD sometimes rapidly emerge. For example, the 19A/ST320 strain was not detected during extensive characterization of pre-PCV7 ABCs isolates (*13*), yet it became the predominant invasive pneumococcal strain during 2005–2009 (*14**,**32*). We have performed comprehensive strain characterization (MLST and WGS) of pediatric (from children <5 years of age) ABCs isolates obtained during 1999, 2001, 2002, 2008, 2009, and 2011–2013 (*6*,*13*) and WGS-based characterization of a large sampling of isolates from all age groups during 1998–2013 (*4**,**6**,**13*; B. Beall, unpub. data).

Before 2015, we detected only 1 isolate of the 35B/ST156 lineage that was recovered during 2012 (*6*). Thus, we feel justified in describing it as newly emergent isolate. A smaller study was recently published (during peer review of this article) that described 78 invasive and 48 noninvasive serotype 35B isolates obtained during 1994–2014 from 8 hospitals in 8 states (*33*). Our data, which included a population-based sampling of 199 35B isolates obtained during 2015–2016, clearly shows the current national predominance of 35B/ST558 and does not support the observation that 35B/ST156 is the major contributor to post-PCV13 antimicrobial-resistant 35B. Both studies noted the initial appearance and emergence of 35B/ST156 in the post-PCV13 period.

This recent identification of the antimicrobial-resistant 35B/ST156 lineage and its subsequent detection within 6 ABCs sites is a cause for concern. The ST156 lineage has shown a remarkable propensity to persist through undergoing serotype-switch events (*12**,**23**,**25**,**32*). The penicillin-resistant 9V/ST156 lineage was the predominant serotype 9V cause of IPD in the United States during the pre-PCV7 era (*13**,**32*). Soon after introduction of PCV7, serotype 9V IPD became rare (*1**,**2*), and 19A became the predominant representative of the ST156 lineage within ABCs (*14**,**32*). After introduction of PCV13, 35B has become the predominant serotype of the ST156 lineage within the United States (B. Beall, unpub. data).

A distinct antimicrobial-susceptible serotype 35B SLV of ST156 (35B/ST162) is included among 35B ABCs of 35B during 2015–2016 by the β-lactam–susceptible PBP type 0:0:0. Thus, our data indicates that >3 independent serotype switches involving the nonvaccine type *cps35B* locus and the broad ST156 clonal complex serving as recipient strains have previously occurred. In this study, we demonstrated that all penicillin-nonsusceptible 35B/ST156 lineage isolates obtained during current ABCs (2015–2016) arose through a single ancestral recombination event. This event was facilitated through detailed analyses of crossover points and comparisons of corresponding regions of all progeny isolates with likely parental 35B/ST558 and 9V/ST156 strains. The genetic plasticity of the ST156 lineage is also highlighted in this study by detection of postserotype switch changes affecting β-lactam resistance (PBP1a type) and capsular serotype (*wciZ* deletion), which is potentially reflective of recent antimicrobial drug pressure and immunologic selection pressure.

An additional 35B variant within a vaccine serotype lineage is shown with ST1092 that is typically associated with serogroup 6 strains (Figure 1). Because these putative 35B switch variants were not detected during extensive strain surveillance before and shortly after conjugate vaccine implementation (*3**,**4**,**13*), it is plausible that these serotype switches occurred after implementation of conjugate vaccine. The observation of a 35B variant within the antimicrobial 15A/ST63 lineage brings the number of serotype switch events generating 35B strains described in this study to 5; (35B/ST11818, 35B/ST156, 35B/ST162 from 2015–2016, and 35B/ST10174 from 2009). Except for the 35B/ST162 variant, these serotype switch events were predicted on the basis of progeny PBP type to involve the 35B/ST558 strain as the *cps35B* donor (online Technical Appendix Table).

Although conjugate vaccines have a history of providing effective and durable protection against IPD (*1**,**2*), the continued emergence and expansion of serotype 35B into different clonal complexes supports continued development of wider spectrum pneumococcal vaccines. Serotype 19A IPD, although relatively uncommon in the pre-PCV7 era, rapidly became the predominant invasive serotype in the post-PCV7 period (*14**,**32**,**34*). Serotype 35B strains have several of the same features that were found among serotype 19A strains before implementation of PCV7 in 2000. These features that could predispose for serotype 35B to continue its increasing trend as a cause of IPD include its lack of inclusion within conjugate vaccine, high carriage rates within children, antimicrobial resistance, clonal expansion, and serotype switching. An experimental 15-valent conjugate vaccine in development includes serotypes 22F and 33F (*35*), which have increased as causes of IPD in the postconjugate vaccine era. Serotypes 15A, 15B, and 23A are expressed by moderately antimicrobial-resistant clones and are not uncommon causes of IPD (*4**,**32*). Although less resistant to β-lactam antimicrobial drugs than 35B/ST558 and 35B/ST156, these strains also present a challenge to address through more encompassing pneumococcal vaccines.

Technical Appendix Additional information on **i**nvasive serotype 35B pneumococci including an expanding serotype switch lineage, United States, 2015–2016.
