# Correction: Vol. 23, No. 6

**DOI:** 10.3201/eid2310.C12310

**Published:** 2017-10

**Keywords:** errata, erratum, correction

In Invasive Serotype 35B Pneumococci Including an Expanding Serotype Switch Lineage, United States, 2015–2016 (S. Chochua et al.), serotype 35B was incorrectly described as the most common cause of invasive pneumococcal disease in children and adults. It is among the most common causes, but not the most common. The article has been corrected online (https://wwwnc.cdc.gov/eid/article/23/6/17-0071_article).

