# Supplementary material for: Invasive Serotype 35B Pneumococci Including an Expanding Serotype Switch Lineage, United States, 2015–2016
Source: Emerg Infect Dis. 2017 Jun;23(6):922–30. doi: 10.3201/eid2306.170071 (PMC5443455; doi:10.3201/eid2306.170071)
Supplement: Technical Appendix — Additional information on invasive serotype 35B pneumococci including an expanding serotype switch lineage, United States, 2015–2016. [file 17-0071-Techapp-s1.pdf]

Article DOI: http://dx.doi.org/10.3201/eid2306.170071

Invasive Serotype 35B Pneumococci Including an Expanding Serotype Switch Lineage, United States, 2015–2016

Technical Appendix

Technical Appendix Table. Accession numbers, whole-genome sequencing pipeline features, and quality metrics of 207 invasive pneumococcal isolates, United States, 2015–2016*

SRA accession no.	Isolate name	State†	Year of isolation	Serotype	pI-1‡	pI-2‡	MLST	PBP type 1a:2b:2x§	Non-PBP resistance determinants¶	No. contigs	N50, bases	Length of longest contig, bases	No. bases in contigs	No. contigs >1K	No. bases in contigs >1K
ERR586423#	2010200750	CA	2009	19A	Yes	No	156	29:12:26	*mef, folAI100L, folPins189*	84	105,726	341,189	2,087,641	39	2,073,476
SAMN05220851	20151623	GA	2015	35B	Yes	No	558	4:7:7	*mef*	345	11,534	52,098	2,066,482	299	2,028,530
SAMN05220873	20151893	GA	2015	35B	Yes	No	558	4:7:7	*mef*	69	104,614	165,383	2,022,450	31	2,010,218
SAMN05220875	20151895	GA	2015	35B	Yes	No	558	4:7:7	*mef*	50	78,901	206,084	2,098,334	40	2,091,749
SAMN05220883	20152130	GA	2015	35B	Yes	No	558	4:7:7	Negative	31	120,620	268,659	2,021,152	25	2,017,441
SAMN05220884	20152131	GA	2015	35B	Yes	No	156	4:12:7	*mef, folAI100L, folPins180*	63	61,342	238,796	2,101,100	57	2,097,051
SAMN05220924	20152247	GA	2015	35B	Yes	No	558	4:7:7	*mef*	32	165,555	329,660	2,023,444	25	2,019,140
SAMN05220932	20152255	GA	2015	35B	Yes	No	558	4:7:7	*mef*	45	91,096	208,104	2,060,658	38	2,056,189
SAMN05220978	20152635	GA	2015	35B	Yes	No	558	4:7:7	*mef*	45	105,615	158,315	2,022,574	36	2,017,138
SAMN05220988	20152649	GA	2015	35B	Yes	No	558	4:7:7	*mef*	63	74,766	267,777	2,055,552	49	2,047,804
SAMN05220990	20152651	GA	2015	35B	Yes	No	558	4:7:7	*mef*	36	158,343	260,315	2,048,689	26	2,042,220
SAMN05220991	20152652	GA	2015	35B	Yes	No	11250	4:14:7	*folPins169*	70	66,332	152,334	2,066,222	60	2,060,214
SAMN05221020	20152694	NM	2015	35B	Yes	No	558	4:7:7	*folPins195*	2255	3,153	35,263	3,471,381	770	2,623,239
SAMN05221036	20152712	NM	2015	35B	Yes	No	558	4:7:7	Negative	51	165,485	264,926	2,021,941	28	2,010,850
SAMN05221054	20152805	GA	2015	35B	Yes	No	558	4:7:7	*mef*	153	28,000	77,453	2,024,387	125	2,008,430
SAMN05221068	20152819	GA	2015	19A	Yes	No	156	8:12:36	*mef, folAI100L, folPins189*	54	79,725	171,901	2,107,451	47	2,103,268
SAMN05221081	20152877	TN	2015	35B	Yes	No	156	4:12:7	*mef, folAI100L, folPins178*	67	69,960	240,680	2,142,873	54	2,134,812
SAMN05221087	20152884	TN	2015	35B	Yes	No	9910	4:12:7	*mef, folAI100L, folPins180*	60	78,563	427,552	2,141,711	45	2,133,089
SAMN05221099	20152896	TN	2015	35B	No	No	558	4:7:7	*mef*	40	105,973	253,714	2,049,350	29	2,042,292
SAMN05221130	20152953	CT	2015	35B	Yes	No	558	4:7:7	*mef*	41	104,752	359,235	2,048,113	33	2,043,373
SAMN05221131	20152954	CT	2015	35B	Yes	No	558	4:7:7	*mef, ParC-S79F*	43	99,836	267,811	2,048,448	34	2,042,394
SAMN05221137	20152960	CT	2015	35B	Yes	No	558	4:7:7	*mef*	50	93,353	225,698	2,052,543	39	2,045,342
SAMN05221153	20153004	NY	2015	35B	Yes	No	558	4:7:7	*mef*	43	106,157	256,689	2,015,996	35	2,011,436
SAMN05221170	20153021	GA	2015	35B	Yes	No	558	4:7:133	*mef*	61	64,424	171,124	2,056,356	55	2,052,821
SAMN05221230	20153206	CO	2015	35B	Yes	No	558	4:7:7	Negative	61	69,265	185,639	2,020,833	53	2,015,716
SAMN05221253	20153229	CO	2015	35B	Yes	No	11254	4:7:7	*folAI100L, folPins169*	54	69,587	141,164	2,067,296	48	2,063,425
SAMN05221268	20153245	NY	2015	35B	Yes	No	558	4:7:7	*mef*	48	100,363	186,047	2,043,861	37	2,038,001
SAMN05221290	20153268	TN	2015	35B	Yes	No	558	4:7:112	*mef*	36	110,217	306,204	2,022,381	30	2,019,060
SAMN05221329	20153308	TN	2015	35B	Yes	No	558	4:7:7	*mef*	56	75,409	139,907	2,025,258	49	2,020,002

SRA accession no.	Isolate name	State†	Year of isolation	Serotype	pI-1‡	pI-2‡	MLST	PBP type 1a:2b:2x§	Non-PBP resistance determinants¶	No. contigs	N50, bases	Length of longest contig, bases	No. bases in contigs	No. contigs >1K	No. bases in contigs >1K
SAMN05221338	20153394	CA	2015	35B	No	No	452	0:0:0	Negative	77	71,399	123,897	2,089,273	50	2,074,421
SAMN05221366	20153422	CA	2015	9V	Yes	No	156	15:12:18	*ermB, folAI100L, folPins178, tetM*	58	74,719	243,646	2,067,218	47	2,061,016
SAMN05221369	20153425	CA	2015	35B	No	No	452	0:0:0	Negative	71	101,781	195,615	2,161,796	37	2,143,676
SAMN05221410	20153478	NM	2015	35B	Yes	No	10493	4:7:7	*mef*	53	78,074	174,938	2,040,592	51	2,039,384
SAMN05221412	20153526	GA	2015	35B	No	No	558	4:7:7	*mef*	48	93,085	210,845	2,051,947	38	2,046,086
SAMN05221447	20153901	MN	2015	35B	Yes	No	558	4:7:7	*mef*	75	51,030	133,733	2,018,673	70	2,015,621
SAMN05221448	20153902	MN	2015	35B	Yes	No	558	4:7:7	*mef*	52	92,130	159,730	2,014,917	42	2,007,904
SAMN05221483	20153937	MN	2015	35B	Yes	No	558	4:7:7	*mef*	45	110,339	267,781	2,022,658	32	2,015,024
SAMN05221491	20153945	MN	2015	35B	Yes	No	558	4:7:7	*mef*	33	165,574	276,997	2,021,333	26	2,017,038
SAMN05221520	20153974	MN	2015	35B	Yes	No	558	4:7:7	Negative	69	74,571	156,076	2,022,222	58	2,014,941
SAMN05221873	20154264	NM	2015	35B	Yes	No	10493	4:7:7	*mef, folAI100L*	38	106,125	297,817	2,051,274	30	2,046,287
SAMN05221920	20154350	NY	2015	35B	Yes	No	558	4:7:7	*mef*	83	48,103	154,090	2,027,137	74	2,020,997
SAMN05221961	20154457	TN	2015	35B	Yes	No	558	4:7:7	*folPins173*	70	75,849	112,748	2,022,694	56	2,016,001
SAMN05221992	20154489	CT	2015	35B	Yes	No	7487	4:49:7	Negative	49	92,283	165,554	2,026,826	40	2,021,564
SAMN05222034	20154575	GA	2015	35B	Yes	No	558	4:7:7	*mef*	29	123,759	268,656	2,016,212	24	2,012,931
SAMN05222063	20154698	MN	2015	35B	Yes	No	558	4:7:7	*mef*	65	71,564	158,172	2,012,692	54	2,005,300
SAMN05222074	20154709	MN	2015	35B	Yes	No	558	4:7:7	*mef*	30	110,276	313,438	2,006,123	23	2,002,203
SAMN05222102	20154740	MN	2015	35B	Yes	No	558	4:7:7	*mef*	49	82,837	206,129	2,007,751	40	2,002,868
SAMN05222113	20154753	MN	2015	31	Yes	No	156	15:12:18	*mef, folAI100L, folPins178*	56	78,904	241,592	2,091,629	43	2,083,767
SAMN05222122	20154762	MN	2015	35B	Yes	No	558	4:7:7	Negative	115	34,829	145,244	2,060,779	98	2,049,243
SAMN05222124	20154764	MN	2015	35B	Yes	No	558	4:7:7	*mef*	40	102,412	344,853	2,001,315	35	1,998,325
SAMN05222138	20154861	CO	2015	35B	Yes	No	558	4:7:7	*mef*	50	91,885	174,021	2,017,466	42	2,012,697
SAMN05222147	20154871	CO	2015	35B	Yes	No	558	4:7:7	Negative	48	86,836	146,223	2,013,425	43	2,010,386
SAMN05222149	20154873	CO	2015	35B	No	No	452	0:0:148	Negative	71	69,780	195,635	2,085,785	50	2,072,719
SAMN05222152	20154876	CO	2015	35B	Yes	No	558	4:7:28	*mef*	47	88,654	221,878	2,017,098	41	2,013,130
SAMN05222170	20154894	CO	2015	35B	Yes	No	558	4:7:7	Negative	56	80,744	260,302	2,045,935	43	2,037,405
SAMN05222174	20154898	CO	2015	35B	Yes	No	558	4:7:7	*mef*	43	120,528	223,043	2,014,603	33	2,008,712
SAMN05222180	20154904	CO	2015	35B	Yes	No	558	4:7:7	Negative	95	71,997	136,148	2,009,843	57	1,995,835
SAMN05222199	20154923	CO	2015	35B	Yes	No	11606	4:7:7	Negative	60	67,755	198,530	2,015,960	55	2,012,635
SAMN05222217	20154941	NY	2015	35B	Yes	No	558	4:7:7	Negative	64	61,235	159,717	2,014,227	58	2,010,063
SAMN05222220	20154980	TN	2015	35B	Yes	No	11584	4:12:7	*mef, folAI100L, folPins178*	62	68,761	174,251	2,138,313	52	2,131,321
SAMN05222224	20154985	TN	2015	35B	Yes	No	7486	4:7:7	*mef*	61	77,881	175,856	2,026,726	53	2,022,345
SAMN05222284	20155252	NM	2015	35B	Yes	No	558	4:7:7	*mef*	55	86,578	170,908	2,038,123	43	2,030,908
SAMN05222300	20155285	CT	2015	35B	Yes	No	558	4:7:7	*mef*	96	48,836	105,501	2,036,455	61	2,025,412
SAMN05222316	20155301	CT	2015	35B	Yes	No	558	4:7:7	*mef*	36	123,831	275,657	2,008,754	27	2,002,993
SAMN05222339	20155324	CT	2015	35B	Yes	No	558	4:7:7	*mef*	126	29,682	73,368	2,027,346	113	2,018,266
SAMN05222355	20155341	NY	2015	35B	Yes	No	558	4:7:7	*mef*	39	105,629	166,148	2,008,199	32	2,003,865
SAMN05222369	20155422	MD	2015	35B	Yes	No	558	4:7:7	*mef*	58	62,697	106,398	2,018,841	55	2,016,970
SAMN05222370	20155424	MD	2015	35B	Yes	No	558	4:7:7	*mef*	98	42,299	117,380	2,056,258	87	2,049,421
SAMN05222399	20155454	MD	2015	35B	Yes	No	11254	4:7:7	*folAI100L, folPins169*	78	48,396	116,466	2,035,836	71	2,031,364
SAMN05222426	20155483	MD	2015	35B	Yes	No	558	4:7:7	*mef, ParC-D83Y*	61	90,884	267,659	2,015,042	31	2,003,664
SAMN05222453	20155510	MD	2015	35B	Yes	No	558	4:7:7	*folPins189*	42	113,849	211,223	2,014,392	28	2,007,669
SAMN05222454	20155511	MD	2015	35B	Yes	No	558	4:7:7	*mef*	59	97,746	220,585	2,019,599	40	2,010,752
SAMN05222473	20155530	MD	2015	35B	Yes	No	558	4:7:7	Negative	99	40,708	108,537	2,058,817	90	2,052,352
SAMN05222480	20155537	MD	2015	35B	Yes	No	558	4:7:7	*mef*	41	93,125	177,099	2,013,666	33	2,009,209

SRA accession no.	Isolate name	State†	Year of isolation	Serotype	pI-1‡	pI-2‡	MLST	PBP type 1a:2b:2x§	Non-PBP resistance determinants¶	No. contigs	N50, bases	Length of longest contig, bases	No. bases in contigs	No. contigs >1K	No. bases in contigs >1K
SAMN05222504	20155562	TN	2015	13	Yes	No	156	15:12:173	*folAI100L, folPins178*	43	111,285	255,659	2,045,236	31	2,039,152
SAMN05222517	20155901	GA	2015	35B	Yes	No	558	4:7:7	*mef*	41	112,540	165,695	2,013,797	30	2,007,676
SAMN05596826	20155922	MN	2015	35B	Yes	No	558	4:7:7	*mef*	32	106,109	325,049	1,999,846	26	1,996,090
SAMN05596827	20155926	MN	2015	35B	Yes	No	156	4:12:7	*mef, folAI100L, folPins178*	49	78,775	279,019	2,126,279	39	2,120,858
SAMN05596828	20155951	MN	2015	35B	Yes	No	558	4:7:7	*mef*	59	73,152	196,224	2,010,083	49	2,003,658
SAMN05222556	20156078	TN	2015	35B	Yes	No	558	4:7:7	Negative	59	82,107	221,409	2,025,411	49	2,020,007
SAMN05222599	20156298	GA	2015	35B	Yes	No	558	4:7:7	*mef*	47	93,019	223,438	2,017,585	33	2,010,494
SAMN05222602	20156301	GA	2015	35B	Yes	No	558	4:7:7	*mef*	45	105,889	262,369	2,011,956	33	2,006,448
SAMN05222619	20156540	CA	2015	35B	Yes	No	558	4:7:7	Negative	42	93,474	260,132	2,013,813	34	2,008,687
SAMN05222660	20156581	CA	2015	35B	No	No	558	4:7:7	*mef*	43	85762	301,048	2,002,644	40	2,000,453
SAMN05222676	20156649	CT	2015	35B	Yes	No	558	4:7:7	*mef*	41	104,825	223,465	2,042,864	32	2,038,004
SAMN05222686	20156659	CT	2015	35B	Yes	No	558	4:7:7	Negative	64	59,402	158,272	2,050,784	57	2,046,464
SAMN05222693	20156666	CT	2015	35B	Yes	No	11603	4:7:7	*mef*	50	91,521	256,674	2,017,334	39	2,011,233
SAMN05222697	20156670	CT	2015	35B	Yes	No	558	4:7:7	*mef*	51	90,896	165,366	2,014,227	34	2,007,629
SAMN05222704	20156677	MD	2015	35B	Yes	No	558	4:7:7	Negative	65	66,752	162,146	2,019,702	58	2,015,338
SAMN05222705	20156678	MD	2015	35B	Yes	No	558	4:7:7	*mef*	48	105,515	221,519	2,018,478	42	2,014,793
SAMN05222711	20156684	MD	2015	35B	No	No	452	0:0:0	*mef*	68	69,921	161,942	2,087,079	47	2,075,378
SAMN05222715	20156689	MD	2015	35B	Yes	No	558	4:7:7	*mef*	37	91,052	279,457	2,016,313	30	2,012,047
SAMN05596829	20156933	MN	2015	35B	Yes	No	558	4:7:7	*mef*	47	77,165	157,740	1,997,705	39	1,992,311
SAMN05596830	20156936	MN	2015	35B	Yes	No	558	4:7:7	Negative	56	79,321	204,697	2,051,711	45	2,045,286
SAMN05596831	20156940	MN	2015	35B	Yes	No	156	4:12:7	*mef, folAI100L, folPins178*	60	65,782	210,473	2,129,301	52	2,124,334
SAMN05222800	20160119	GA	2015	35B	Yes	No	156	4:12:7	*mef, folAI100L, folPins178*	74	58,812	117,153	2,134,217	66	2,129,337
SAMN05222828	20160273	TN	2015	35B	Yes	No	558	4:7:7	*mef,parC-D83Y, gyrA-S81Y*	138	27,871	91,211	2,035,655	128	2,028,330
SAMN05222863	20160308	GA	2015	35B	Yes	No	558	4:7:7	Negative	46	101,661	165,518	2,045,468	38	2,040,619
SAMN05222880	20160528	MD	2015	35B	Yes	No	558	4:7:7	*mef*	85	40,578	102,802	2,055,955	78	2,051,459
SAMN05222885	20160534	MD	2015	35B	Yes	No	11604	4:7:7	*mef*	50	89,698	171,807	2,014,102	37	2,008,156
SAMN05222910	20160559	TN	2015	35B	Yes	No	558	4:7:7	*mef*	63	80,228	167,817	2,004,803	47	1,995,742
SAMN05222922	20160571	CT	2015	35B	Yes	No	558	4:7:7	*mef*	51	76,272	227,736	2,017,530	43	2,013,125
SAMN05222929	20160578	CT	2015	35B	Yes	No	10493	4:7:7	*mef*	74	81,944	305,405	2,071,800	47	2,060,517
SAMN05222937	20160586	CT	2015	35B	Yes	No	558	4:7:7	*mef*	71	63,833	139,228	2,013,553	59	2,006,086
SAMN05222969	20160886	GA	2015	35B	Yes	No	558	4:7:7	*mef*	114	35,631	159,040	2,069,781	103	2,061,803
SAMN05222989	20160906	NM	2015	35B	Yes	No	558	4:7:7	Negative	47	87,665	172,854	2,013,642	40	2,009,199
SAMN05222997	20160914	NM	2015	35B	Yes	No	10493	4:7:7	*mef*	54	82,019	179,360	2,080,791	51	2,078,481
SAMN05223002	20160919	NM	2015	35B	Yes	No	1092	6:7:36	*folAI100L, folPins195*	115	36,416	114,401	2,161,903	104	2,154,336
SAMN05223093	20161195	NY	2015	35B	Yes	No	156	4:12:7	*mef, folAI100L, folPins178*	67	60,715	149,428	2,132,963	55	2,126,325
SAMN05596832	20161250	MN	2015	35B	Yes	No	558	4:7:7	*mef*	63	74,782	134,247	2,022,806	53	2,017,060
SAMN05596833	20161257	MN	2015	35B	Yes	No	558	4:7:7	*mef*	60	78,703	113,175	2,011,397	53	2,006,497
SAMN05596835	20161304	MN	2015	35B	Yes	No	558	4:7:7	*mef*	58	83,268	230,802	2,009,412	47	2,002,330
SAMN05596836	20161312	MN	2015	35B	Yes	No	558	4:7:7	*mef*	168	25,368	105,651	2,070,391	150	2,058,010
SAMN05596837	20161318	MN	2015	35B	Yes	No	558	4:7:7	*mef*	62	68,201	194,581	2,010,256	53	2,004,559
SAMN05223133	20161390	GA	2015	35B	Yes	No	558	4:7:7	*mef*	39	112,692	232,100	2,016,345	31	2,011,820
SAMN05223145	20161402	GA	2015	35B	Yes	No	558	4:7:7	*mef*	43	90,976	173,428	2,048,722	36	2,044,540

SRA accession no.	Isolate name	State†	Year of isolation	Serotype	pI-1‡	pI-2‡	MLST	PBP type 1a:2b:2x§	Non-PBP resistance determinants¶	No. contigs	N50, bases	Length of longest contig, bases	No. bases in contigs	No. contigs >1K	No. bases in contigs >1K
SAMN05223156	20161413	GA	2015	35B	Yes	No	156	4:12:7	*mef, folAI100L, folPins178*	55	70,259	279,680	2,130,702	47	2,125,944
SAMN05223181	20161606	CT	2015	9V	Yes	No	156	15:12:18	*mef, folAI100L, folPins178, tetM*	55	69,396	238,719	2,117,477	46	2,111,818
SAMN05223194	20161619	CT	2015	35B	Yes	No	558	4:7:7	*mef*	37	120,271	173,384	2,014,607	28	2,009,100
SAMN05223196	20161621	CT	2015	35B	Yes	No	558	4:7:7	*mef*	52	110,264	221,047	2,053,911	35	2,045,003
SAMN05223197	20161622	CT	2015	35B	Yes	No	558	4:7:7	*mef*	38	93,266	268,577	2,007,538	29	2,002,683
SAMN05223202	20161627	CT	2015	35B	Yes	No	558	4:7:7	*mef*	37	109,078	329,557	2,008,734	31	2,005,184
SAMN05223206	20161631	CT	2015	35B	Yes	No	558	4:120:7	*mef*	38	112,348	283,017	2,051,468	29	2,045,940
SAMN05223222	20161647	MD	2015	35B	No	No	11818	4:31:114	*ermB, folPins195, tetM*	56	117,980	273,832	2,093,564	35	2,083,044
SAMN05223244	20161669	MD	2015	35B	Yes	No	6961	4:7:7	Negative	46	89,773	173,235	2,082,077	36	2,077,396
SAMN05223249	20161674	MD	2015	35B	Yes	No	558	4:7:7	*mef*	52	93,157	157,673	2,021,717	41	2,016,287
SAMN05596838	20161697	MD	2015	35B	Yes	No	558	4:7:7	*mef*	76	49,448	128,234	2,023,535	69	2,018,951
SAMN05596839	20161763	TN	2015	35B	Yes	No	156	4:12:7	*mef, folAI100L, folPins178*	74	61,347	118,236	2,139,347	61	2,130,933
SAMN05596840	20161772	TN	2015	35B	Yes	No	558	4:7:7	*mef*	35	94,121	214,007	2,007,894	30	2,004,814
SAMN05596841	20161796	TN	2015	35B	Yes	No	558	4:7:7	*mef*	50	104,680	260,238	2,015,939	34	2,009,679
SAMN05596842	20161806	TN	2015	35B	Yes	No	558	4:7:7	*mef*	53	70,235	170,203	2,017,465	43	2,011,036
SAMN05596843	20161992	CA	2015	35B	No	No	452	0:0:0	Negative	93	68,407	129,046	2,125,843	61	2,109,508
SAMN05596848	20162335	TN	2015	35B	Yes	No	558	4:7:7	*mef*	79	81,310	165,420	2,040,006	51	2,031,050
SAMN05596849	20162342	TN	2015	35B	Yes	No	156	4:12:7	*mef, folAI100L, folPins178*	164	38,112	116,941	2,132,394	90	2,109,381
SAMN05596853	20162573	NM	2015	35B	Yes	No	558	4:7:7	*mef*	46	95,570	165,406	2,005,856	35	2,000,856
SAMN05751705	20162860	TN	2015	35B	Yes	No	558	102:7:7	*mef*	52	92,709	202,610	1,998,583	34	1,990,417
SAMN05596864	20163113	MN	2015	35B	Yes	No	558	4:7:7	*mef*	50	89,836	221,579	2,017,514	40	2,012,326
SAMN05596873	20163679	TN	2015	35B	Yes	No	11584	4:12:7	*mef, folAI100L, folPins178*	48	79,119	492,175	2,132,330	40	2,127,528
SAMN05617281	20164527	CO	2015	9V	Yes	No	156	15:12:18	*mef, folAI100L, folPins178*	76	105,812	265,417	2,096,832	45	2,082,189
SAMN05596844	20162014	CA	2016	35B	Yes	No	558	4:7:7	*mef, folPins186*	35	150,817	377,182	2,013,395	26	2,007,686
SAMN05596845	20162040	GA	2016	35B	Yes	No	558	4:7:7	*mef*	44	106,033	260,232	2,016,925	30	2,008,717
SAMN05596846	20162045	GA	2016	35B	Yes	No	558	4:7:7	*mef*	60	84,502	146,825	2,013,691	45	2,006,344
SAMN05596847	20162312	NY	2016	35B	Yes	No	558	4:7:7	*mef*	34	108,278	267,785	2,008,023	26	2,003,188
SAMN05596850	20162366	GA	2016	35B	Yes	No	11866	4:7:7	*mef*	42	105,288	165,485	2,012,415	34	2,007,634
SAMN05596851	20162388	GA	2016	35B	Yes	No	558	4:7:7	*mef*	32	123,819	268,582	2,014,483	23	2,009,884
SAMN05596852	20162390	GA	2016	35B	Yes	No	156	4:12:7	*mef, folAI100L, folPins178*	53	69,911	361,463	2,129,840	42	2,123,871
SAMN05596854	20162889	TN	2016	35B	Yes	No	156	4:12:7	*mef, folAI100L, folPins178*	61	63,143	149,031	2,092,555	50	2,086,601
SAMN05596856	20162912	CT	2016	35B	Yes	No	156	4:12:7	*mef, folAI100L, folPins178*	49	90,635	216,786	2,130,046	43	2,126,635
SAMN05596857	20162917	CT	2016	35B	Yes	No	558	4:7:7	Negative	70	63,408	205,652	2,049,848	58	2,042,132
SAMN05596858	20162920	CT	2016	35B	No	No	452	0:0:0	Negative	78	73,710	186,612	2,085,665	50	2,071,103
SAMN05596859	20162941	MD	2016	35B	Yes	No	558	4:7:7	*mef*	44	106,088	253,376	2,043,264	34	2,037,582
SAMN05596860	20162948	MD	2016	35B	Yes	No	558	4:7:7	Negative	75	63,449	150,746	2,043,703	55	2,035,188
SAMN05596861	20162969	MD	2016	35B	Yes	No	558	4:7:7	*mef*	57	75,713	175,536	2,015,950	49	2,011,333
SAMN05596862	20162972	MD	2016	35B	Yes	No	558	106:7:7	Negative	54	77145	178,983	2,014,160	42	2,007,683

SRA accession no.	Isolate name	State†	Year of isolation	Serotype	pI-1‡	pI-2‡	MLST	PBP type 1a:2b:2x§	Non-PBP resistance determinants¶	No. contigs	N50, bases	Length of longest contig, bases	No. bases in contigs	No. contigs >1K	No. bases in contigs >1K
SAMN05596863	20163008	NY	2016	35B	Yes	No	558	4:123:7	*mef*	38	112,805	313,544	2,006,543	26	2,001,014
SAMN05596865	20163194	MN	2016	35B	Yes	No	558	4:7:7	*mef*	56	89,735	262,271	2,012,685	38	2,004,614
SAMN05596866	20163213	MN	2016	35B	Yes	No	558	4:7:7	*mef*	58	91,575	256,172	2,057,438	41	2,048,656
SAMN05596867	20163215	MN	2016	35B	Yes	No	558	4:7:7	*mef*	38	104,755	221,865	2,008,017	32	2,004,469
SAMN05596868	20163410	NM	2016	35B	No	No	452	0:0:0	Negative	68	92,976	206,750	2,128,641	42	2,115,150
SAMN05596869	20163480	GA	2016	35B	Yes	No	558	4:7:7	*mef*	33	106,001	313,410	2,013,072	27	2,009,308
SAMN05596870	20163509	GA	2016	35B	Yes	No	558	4:7:7	*folAI100L*	43	86,577	223,544	2,049,887	37	2,046,110
SAMN05596871	20163642	TN	2016	35B	Yes	No	156	4:12:7	*mef, folAI100L, folPins178*	52	90,465	210,435	2,127,197	39	2,120,744
SAMN05596872	20163669	TN	2016	35B	Yes	No	558	4:7:7	*mef*	34	120,612	268,637	2,015,492	28	2,011,925
SAMN05596874	20163701	OR	2016	35B	No	No	452	0:0:0	Negative	66	94,,801	195,539	2,081,512	40	2,068,754
SAMN05596875	20163728	NY	2016	35B	Yes	No	558	4:7:7	*mef*	478	26,166	115,452	2,054,312	137	1,963,751
SAMN05596876	20164142	NM	2016	35B	Yes	No	162	0:0:0	Negative	115	56,676	265,920	2,102,021	59	2,082,245
SAMN05596877	20164157	GA	2016	35B	Yes	No	10493	4:7:7	*mef*	44	93,170	294,685	2,038,238	33	2,033,518
SAMN06215793	20164170	GA	2016	35B	Yes	No	12854	4:7:7	*mef*	76	63,877	173,194	2,058,373	61	2,051,342
SAMN06215794	20164318	MN	2016	35B	Yes	No	558	4:7:7	*mef*	46	104,615	205,294	2,010,923	33	2,004,779
SAMN06215795	20164323	MN	2016	35B	Yes	No	558	4:7:7	*mef*	88	66,808	116,805	2,031,386	52	2,016,536
SAMN06215796	20164352	MD	2016	35B	No	No	10493	4:7:7	*mef*	74	68,187	116,318	2,028,063	53	2,019,108
SAMN06215797	20164372	MD	2016	35B	Yes	No	10493	4:7:7	*mef*	128	46,801	123,721	2,039,754	80	2,023,641
SAMN06215798	20164387	MD	2016	35B	Yes	No	156	4:12:7	*mef, folAI100L, folPins178*	137	48,881	150,191	2,098,651	84	2,080,158
SAMN06215799	20164405	TN	2016	35B	Yes	No	2082189	4:7:7	*mef*	3217	3,817	33,659	2,279,413	359	1,568,218
SAMN06215800	20164408	TN	2016	35B	Yes	No	12921	4:11:7	*mef, folAI100L, folPins178*	97	69,811	147,008	2,126,987	57	2,111,689
SAMN06215801	20164453	CT	2016	35B	No	No	452	0:0:0	Negative	123	50,059	120,756	2,083,262	71	2,062,939
SAMN06215802	20164455	CT	2016	35B	Yes	No	558	4:7:7	Negative	78	78,527	160,641	2,017,615	50	2,005,477
SAMN06215803	20164456	CT	2016	35B	Yes	No	558	4:7:7	*mef*	103	45,263	95,304	2,020,183	79	2,010,278
SAMN06215804	20164465	CT	2016	35B	Yes	No	558	4:7:7	*mef*	70	63,940	147,951	2,019,143	54	2,012,182
SAMN06215805	20164476	CT	2016	35B	Yes	No	558	4:7:7	*mef*	93	63,483	182,215	2,056,675	56	2,043,534
SAMN06215806	20164485	CT	2016	35B	Yes	No	558	4:7:7	Negative	87	63,417	112,701	2,013,264	60	2,002,395
SAMN06215807	20164538	CO	2016	35B	Yes	No	558	4:135:7	*folAI100L, folPins169*	111	47,839	125,940	2,013,053	79	2,003,477
SAMN06215808	20164579	CO	2016	35B	Yes	No	558	4:7:7	*folAI100L, folPins180*	108	54,780	107,696	2,011,176	66	1,998,980
SAMN06215809	20164584	CO	2016	35B	Yes	No	558	4:7:7	*folAI100L, folPins169*	53	94,840	237,216	2,014,730	36	2,007,411
SAMN06215810	20165166	OR	2016	35B	Yes	No	558	4:7:7	*mef*	167	35,168	85,353	2,013,744	95	1,993,804
SAMN06215811	20165435	NY	2016	35B	Yes	No	558	4:7:7	*mef*	114	49,620	165,048	1,997,966	59	1,984,526
SAMN06215812	20165446	TN	2016	35B	Yes	No	558	4:7:7	*mef*	52	106,037	239,738	2,021,148	31	2,012,234
SAMN06215813	20165460	TN	2016	35B	Yes	No	558	4:7:7	Negative	81	78,421	140,820	2,018,764	51	2,006,385
SAMN06215814	20165513	GA	2016	35B	Yes	No	558	4:7:7	*mef*	58	91,406	222,962	2,051,473	43	2,043,757
SAMN06215815	20165562	MN	2016	35B	Yes	No	558	4:7:7	*mef*	70	89,761	201,707	2,016,051	40	2,003,973
SAMN06215816	20165585	MN	2016	35B	Yes	No	558	4:7:7	*mef*	99	51,117	165,382	2,013,853	62	2,000,883
SAMN06215817	20165590	MN	2016	35B	Yes	No	558	4:7:7	*mef*	116	49,676	165,343	2,019,643	76	2,005,318
SAMN06215818	20165613	MN	2016	35B	Yes	No	558	4:139:7	*mef*	61	81,988	172,386	2,039,499	46	2,031,802
SAMN06215819	20165637	MN	2016	35B	Yes	No	558	4:7:7	*mef*	51	82,907	202,647	2,043,980	40	2,038,852
SAMN06215820	20165685	MN	2016	35B	Yes	No	12883	4:7:7	*folAI100L*	57	68,109	253,901	2,018,465	43	2,012,245
SAMN06215821	20165915	CT	2016	35B	Yes	No	12885	4:7:7	*mef*	67	80,988	261,406	2,019,225	46	2,011,232
SAMN06215822	20165986	NY	2016	35B	Yes	No	558	4:7:7	*mef*	84	78,417	165,496	2,086,489	51	2,072,015
SAMN06215823	20165996	CT	2016	35B	Yes	No	558	4:120:7	*mef*	65	76,906	162,547	2,051,848	46	2,043,512

SRA accession no.	Isolate name	State†	Year of isolation	Serotype	pI-1‡	pI-2‡	MLST	PBP type 1a:2b:2x§	Non-PBP resistance determinants¶	No. contigs	N50, bases	Length of longest contig, bases	No. bases in contigs	No. contigs >1K	No. bases in contigs >1K
SAMN06215824	20166006	MD	2016	9V	Yes	No	156	15:12:228	*mef, folAI100L, folPins178*	49	104,699	567,749	2,048,506	32	2,038,541
SAMN06215825	20166007	MD	2016	35B	Yes	No	558	4:7:7	*folAI100L*	34	144,034	329,701	2,048,856	25	2,043,137
SAMN06215826	20166027	MD	2016	35B	Yes	No	558	4:7:7	Negative	40	108,014	257,659	2,002,985	31	1,998,164
SAMN06215827	20166030	MD	2016	35B	Yes	No	12922	4:142:7	Negative	44	105,529	221,286	2,011,129	33	2,005,703
SAMN06215828	20166031	MD	2016	35B	Yes	No	558	4:7:7	*mef*	41	98,102	268,599	2,024,426	33	2,019,673
SAMN06215829	20166040	MD	2016	35B	Yes	No	558	4:7:7	Negative	33	120,367	282,949	2,012,657	28	2,009,526
SAMN06215830	20166079	GA	2016	35B	Yes	No	558	4:7:7	*mef*	32	105,548	237,002	2,010,391	28	2,008,033
SAMN06215831	20166343	OR	2016	35B	No	No	452	0:0:0	Negative	65	64,765	181,330	2,073,513	47	2,064,230
SAMN06555337	20166603	MN	2016	35B	Yes	No	558	4:7:7	*mef*	70	88,319	173,091	2,004,297	39	1,994,204

*MLST, multilocus sequence type; N50, average length of contigs; PBP, penicillin-binding protein; SRA, sequence read archive.
†CA, California; CO, Colorado; CT, Connecticut; GA, Georgia; MD, Maryland; MN, Minnesota; NM, New Mexico; NY, New York; OR, Oregon; TN, Tennessee.
‡Pilus subunit pI-1 and pI-2 genes.
§See Li et al. (*1*) and MIC correlates for PBP types (http://www.cdc.gov/streplab/mic-tables.html).
¶For a description of whole-genome sequence–based bioinformatic pipeline for deduction of all features shown, see Li et al. (*1*) and Metcalf et al. (*2,3*). See Figure 1 in Metcalf et al. (*3*) for a description of *folP* insertions (*folPins178*, *folP189*).
#European Nucleotide Archive accession number.

References

1. Li Y, Metcalf BJ, Chochua S, Li Z, Gertz RE Jr, Walker H, et al. Penicillin-binding protein transpeptidase signatures for tracking and predicting β-lactam resistance levels in *Streptococcus pneumoniae*. MBio. 2016;7:e00756–16. PubMed http://dx.doi.org/10.1128/mBio.00756-16

2. Metcalf BJ, Gertz RE Jr, Gladstone RA, Walker H, Sherwood LK, Jackson D, et al.; Active Bacterial Core surveillance team. Strain features and distributions in pneumococci from children with invasive disease before and after 13-valent conjugate vaccine implementation in the USA. Clin Microbiol Infect. 2016;22:60.e9–29. PubMed http://dx.doi.org/10.1016/j.cmi.2015.08.027

<jrn>3. Metcalf BJ, Chochua S, Gertz RE Jr, Li Z, Walker H, Tran T, et al.; Active Bacterial Core surveillance team. Using whole genome sequencing to identify resistance determinants and predict antimicrobial resistance phenotypes for year 2015 invasive pneumococcal disease isolates recovered in the United States. Clin Microbiol Infect. 2016;22:1002.e1–8. PubMed http://dx.doi.org/10.1016/j.cmi.2016.08.001</jrn>
